# HPV vaccination as preventive approach for recurrent respiratory papillomatosis - a 22-year retrospective clinical analysis

**DOI:** 10.1186/s12879-018-3260-0

**Published:** 2018-07-24

**Authors:** Paul Stefan Mauz, Fabian Axel Schäfer, Thomas Iftner, Phillipp Gonser

**Affiliations:** 1 Department for Otolaryngology, Head and Neck Surgery, University Hospital of Tübingen, Eberhard Karls University Tübingen, Elfriede-Aulhorn-Str 5, DE-72076 Tübingen, Germany; 2 Eberhard Karls University Tübingen, DE-72076 Tübingen, Germany; 3 Division of Experimental Virology, Institute for Medical Virology, University Hospital of Tübingen, Eberhard Karls University Tübingen, DE-72076 Tübingen, Germany

**Keywords:** Recurrent respiratory papillomatosis, Human papillomavirus, Treatment approach, Microdebrider, Gardasil®, Vaccination, Cidofovir®

## Abstract

**Background:**

Recurrent respiratory papillomatosis (RRP) is a rare, benign disease of the aerodigestive tract, especially the larynx, caused by infection with the human papillomavirus (HPV) types 6 or 11. Current management focuses on surgical debulking with microdebrider of papillomatous lesions with or without concurrent adjuvant therapy, e.g. Cidofovir®. This retrospective study evaluates the results of patients treated at a department of the university clinic between 1990 and 2012 and compares the results of the conventional treatment with a new treatment approach using adjuvant vaccination with Gardasil®.

**Methods:**

A retrospective Kaplan Maier analysis of *n* = 24 patients diagnosed and treated with RPR was performed. The records were reviewed for gender, age at the time of first manifestation of disease and time to recurrence.

**Results:**

Only *n* = 2 (15.4%) of the *n* = 13 vaccinated patients developed a recurrence of the disease after a mean time of 54.9 months (SD: 9.5 months). All patients who were not vaccinated (*n* = 11; 100%) developed a relapse after a mean time of 12.3 months (SD: 9.72 months).

**Conclusion:**

We propose that adjuvant HPV vaccination with Gardasil® might have a preventive effect in RRP by occluding new papilloma formation.

## Background

Recurrent respiratory papillomatosis (RRP) is a rare disease affecting children and adults. The wart-like papillomas are caused by an infection with human papilloma viruses (HPV), the larynx being the most frequent localization. The morbidity of RRP is due to its high tendency of recurrence leading to repeated hospitalization with an enormous socioeconomic impact. The course of the RRP is difficult to predict as some patients can be cured whilst in other cases the infection may recur. A recurrence rate of 71.9% in juvenile papillomatosis and 22.8% in adults leads to the necessity of repeated operative interventions for years [1–3]. The cost of a patient suffering from RRP is estimated to be 60,000 to 470,000 USD [4]. Annually, RRP results in 15,000 surgical procedures causing annual cost of 150 million USD in the US [5]. In three to 7 % of RRP patients malignant propgression of the papillomas can be observed [6–8].

The treatment goals, besides virus elimination and curing the patient, are based on the reduction of papillomas, airway protection, prevention of spread and reduction of hospitalization [9, 10].

The gold standard treatment option is the surgical ablation of the papillomas with approximately 20% of patients requiring adjuvant drug therapy in addition to surgical treatment. In the adjuvant therapy of RRP Cidofovir® is one of the most commonly applied medications as it probably blocks virus replication by inducing apoptosis due to DNA damage [11, 12]. In the United States Cidofovir® has only been used intravenously to treat cytomegalovirus-induced retinitis in patients with acquired immunodeficiency syndrome. However, the therapeutic benefit in the treatment of RRP by means of intralesional injection is well documented [13–16]. In addition, a prophylactic vaccination against HPV types 6 and 11 is promising in the prevention of RRP. Currently, three vaccines are approved for the prevention of HPV infection of the anogenital tract: the bivalent vaccine Cervarix® (Glaxo-Smith-Kline), the tetravalent vaccine Gardasil® (Merck) and from 2016 on the nonavalent vaccine Gardasil 9 ® (Merck). The efficacy of Gardasil® is 100% against precancerous cervical lesions induced by HPV type 16 and type 18, 100% against genital warts caused by HPV 6/11 as well as 95% against highly differentiated vaginal neoplasia [17].

Although vaccination of patients with RRP has been discussed for years [18, 19], only anecdotal reports describe the promising therapeutic effect achieved by a Gardasil® immunization [20, 21].

The scope of this retrospective study is to evaluate the clinical results in patients diagnosed with RRP between 1990 and 2012 treated with microdebrider, intralesional Cidofovir® injection and adjuvant vaccination with Gardasil® (intramuscular injection of three doses: day 0, after 8 weeks and after 6 months) in order to investigate the effectiveness of this therapeutic approach in contrast to the conventional treatment without adjuvant vaccination.

## Methods

This study was carried out according to the STROBE guidelines [22] and approval of the local ethics committee was obtained.

A retrospective Kaplan Meier analysis of all patients diagnosed with and treated for RRP at the university clinic between 1990 and 2012 was performed. All patients received surgical treatment with microdebridement of the papillomatous lesions and intralesional Cidofovir® injection. In patients who had agreed into the off-label use of Gardasil® the adjuvant vaccination was performed with intramuscular injection of three doses: day 0, after 8 weeks and after 6 months. There was no scientific intent. The records were reviewed for gender, age at the time of first manifestation of the disease and time to recurrence. In patients not vaccinated with Gardasil®, the time to the first relapse was considered; in vaccinated patients, the first relapse was assessed after the immunization had been completed. Blood analyses were taken and INNO-LiPA HPV Genotyping Extra® test (FujiReBio) was used to determine the human papillomavirus type.

**Fig. 1:**
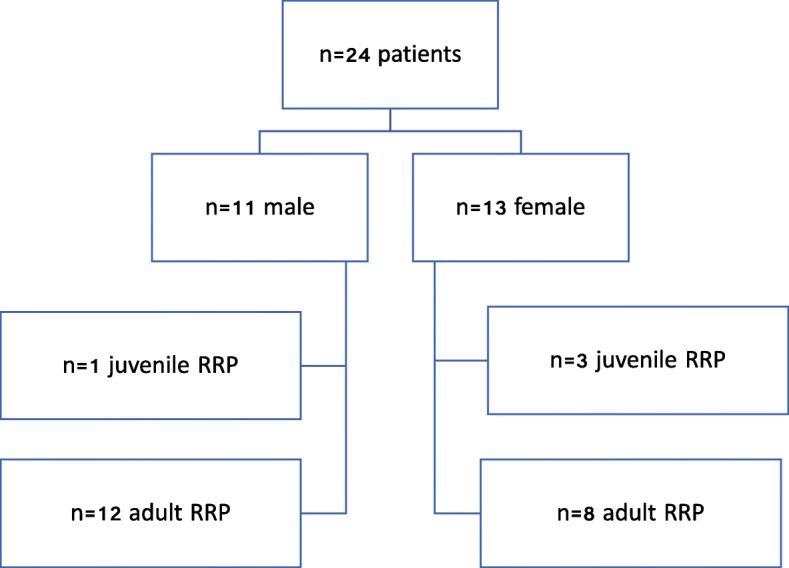
Flow chart showing the number of patients with adult and juvenile recurrent respiratory papillomatosis included in the study

The patient population consisted of *n* = 24 patients (11 female and 13 male). Patients younger than 12 years (*n* = 4, 3 female and 1 male) suffered from juvenile RRP according to the definition of Larson et al. [23]. Thus, *n* = 20 patients represented the group of adult RRP (8 female and 12 male) (Fig. 1).

Statistical evaluation of all data was performed using Microsoft Excel and IBM SPSS Statistics 20 (SPSS Inc., IBM Company, Chicago, IL). For quantitative data the arithmetic mean, minimum (min) and maximum (max), as well as standard deviation (SD) were calculated.

A two-sided log-rank test was used for comparison of the recurrence rates of vaccinated and non-vaccinated patients. A value of *p* < 0.05 was considered to indicate statistically significant differences. An alpha adjustment for multiple tests was not performed, thus the results have to be seen as explorative and descriptive.

## Results

**Fig. 2:**
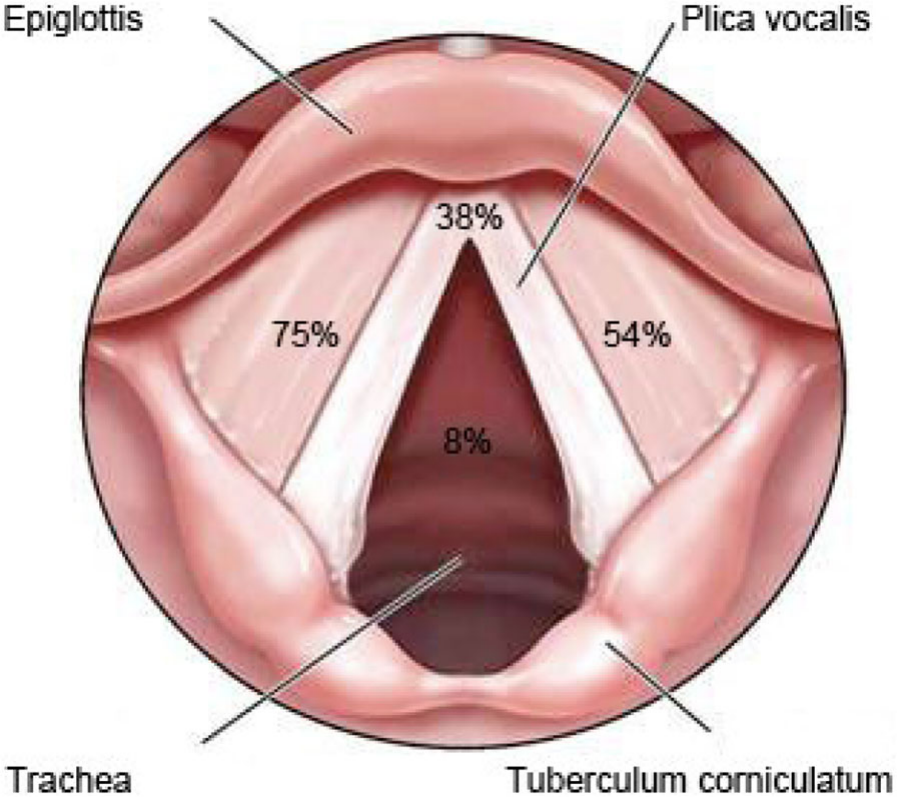
Graphic showing the location of laryngeal manifestation of the RRP in per cent [48]

Between 1990 and 2012 *n* = 24 patients with RRP were treated in our clinic. *N* = 13 (54%) patients were treated with microdebridement, intralesional injection of Cidofovir® and adjuvant vaccination with Gardasil® (Fig. 1), *n* = 11 (46%) patients were treated with conventional microdebridement and intralesional Cidofovir® injection. The mean age at first manifestation was 2.97 years in juvenile RRP (min: 2 years, max: 5 years; SD: 1.46 years) and 48.36 years in adult RRP (min: 15 years, max: 73 years; SD: 17.91 years). Figure 2 shows the locations of laryngeal manifestations of the RRP. Table 1 shows the genotypic characterization of the HPV types in our patient population.

**Table 1:** Table showing the number and percentage of HPV types in our patient population

Genotypic characterization of HPV in our patient population		
HPV type	*n* = number of patients (per cent %)	
	juvenile RRP	adult RRP
type 6	1 (25%)	10 (50%)
type 11	1 (25%)	4 (20%)
type 6/11	1 (25%)	2 (10%)
type 6/16	1 (25%)	–
type 16	–	2 (10%)
type 6/52	–	1 (5%)
type 16/18	–	1 (5%)
total	4	20

**Fig. 3:**
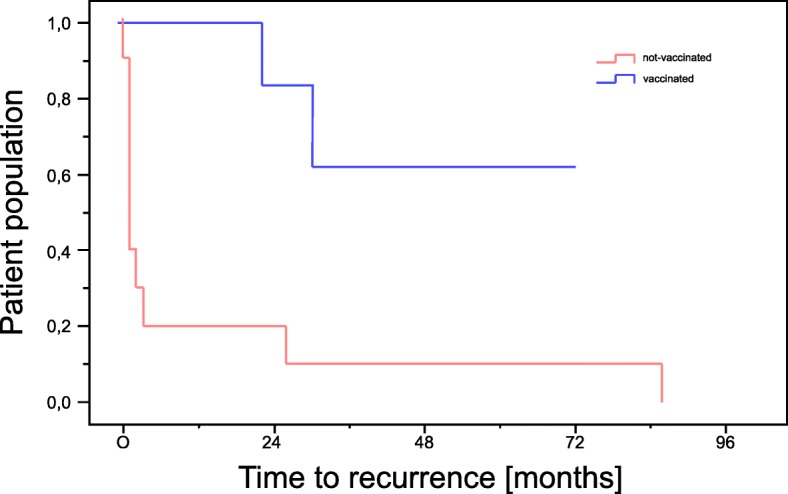
Kaplan Meier chart showing the time to recurrence of the disease in vaccinated and not-vaccinated patients

Of the 13 patients vaccinated only *n* = 2 (15.4%) developed a recurrence of the disease, whereas all patients who were not vaccinated (*n* = 11; 100%) developed a recurrence during the maximum follow-up time of 86 months. The mean time to first recurrence was 12.3 months (SD: 9.72 months) in unvaccinated patients, and 54.9 months (SD: 9.5 months) in the two vaccinated patients (Fig. 3). Time to recurrence was significantly shorter in non-vaccinated patients than in the vaccinated population (log-rank test, *p* < 0.001). Mean number of operations necessary to treat the disease in unvaccinated patients and the *n* = 2 patients with local recurrence was 15.25 (SD: 11.4, min 5, max 27) in juvenile RRP and 4.8 (SD: 3.95, min 1, max 18) in adult RRP.

## Discussion

We retrospectively evaluated the clinical results of 24 patients with RRP treated in our clinic between 1990 and 2012. In 11 patients (46%) therapy consisted of microdebridement and intralesional Cidofovir® injection, while 13 patients (53%) received additional vaccination with Gardasil®.

Even though the number of patients included in this retrospective study is limited, there are currently no studies comparable in cohort-size available in today’s literature.

In our patient population HPV type 6 dominated in 11 patients (45.8%) while HPV type 11 was found in 5 cases (20.8%). The distribution of HPV types in our study is consistent with the results of most previous reports demonstrating HPV type 6 and 11 isolations in 50–100% [24–31] with HPV types 16 and 18 being the second frequent cause for RRP [32–35].

Uloza et al. report a recurrence rate of 71.9% for juvenile papillomatosis and 22.8% for adult RRP [2]. They also show that the interval between relapses is 1 month to 10 years (mean 1.9 years) for the juvenile and 6 months to 9 years (mean 3.2 years) for adult RRP, respectively. Our results show, that only *n* = 2 (15.4%) of the vaccinated patients developed a recurrence of the disease after mean 54.9 months (SD: 9.5 months), whereas all patients who were not vaccinated (*n* = 11; 100%) developed a relapse after a mean time of 12.3 months (SD: 9.72 months), which shows a statistically significant difference in time to relapse (log-rank test, *p* < 0.001).

The basis of RRP therapy is surgical removal of the papillomas with either microdebrider or different types of lasers. We prefer microdebrider over laser-surgical techniques for ablation of the papillomas as the infected tissue is directly removed and the curved construction of the instrument permits a precise treatment of deeper laryngeal regions. Considering a number of about 4 operations necessary in the first year after diagnosis it becomes clear that a gentle and the most precise surgical approach is of great importance [36].

The therapeutic benefit of treating RRP by intralesional injection of Cidofovir® was demonstrated in many studies [13–15]. Chadha et al. summarized the literature on the treatment of RRP with Cidofovir® and concluded that in more than 80% of patients either complete or at least partial healing occurred [37]. A major advantage of intralesional injection is that locally high levels of the active compound are achieved without significantly increasing the plasma level of the drug [38]. When applying Cidofovir® the adverse effects such as nephrotoxicity, bone marrow damage, iritis and uveitis must always be considered. In vitro experiments have shown that Cidofovir ® provokes DNA damage that might promote malignant progression [39].

Currently three HPV vaccines are available. Cervarix ® (Glaxo-Smith-Kline) is a bivalent vaccine against the L1 protein of the HPV types 16 and 18. The HPV types 16 and 18 are the most common viruses causing cervical carcinoma. This vaccine however does not affect HPV types 6 and 11. Gardasil ® (Sanofi Pasteur-MSD / Merck) which we used in our patient population is a quadrivalent vaccine based on virus-like particles (VLP), from the main structural protein L1 from four different viruses thus immunizing against HPV types 6, 11, 16 and 18. Gardasil 9 ® (Sanofi Pasteur-MSD / Merck) is available since 2016 and protects against HPV types 6, 11, 16, 18, 31, 33, 45, 52, und 58.

Numerous clinical studies have shown the safety and effectiveness of Gardasil ® in gynecological patients, and even though Gardasil ® does not cure an existing HPV infection it has been shown to induce a strong humoral immune reaction, which is up to 100-fold stronger than after a natural infection [40, 41]. In a recently published prospective study Novacovic et al. could show a statistically significant decline of the incidence of juvenile RRP after implementation of a national HPV vaccination program with Gardasil ® in Australia from 0.16 per 100,000 in 2012 to 0.02 per 100,000 in 2016 (*p* = 0.034) [42]. Above all, the results suggest that HPV vaccination results in high titers of neutralizing antibodies in individuals showing only a low level of antibody formation after natural infection [43] and by that may help to reduce recurrence or spread of the disease after surgical therapy [44]. While in our study population unvaccinated patients developed a recurrence after 12.3 months, we could extend this time to 54.3 months by vaccinating with Gardasil ®, which is consistent with the results of the case reports of Förster and Mudry [20, 21]. Tjon Pian Gi et al. performed a retrospective observational study in six RRP patients who received a quadrivalent HPV vaccine and measured HPV seroreactivity. Multiplex HPV serology was used to determine HPV- specific antibodies pre- and post-vaccination. Patients did not receive any adjuvant therapy 1 month before, neither during or after vaccination. They found that the quadrivalent HPV vaccine resulted in increased seroreactivity against the causal HPV in every patient [45].

The underlying mechanism of the vaccine’s apparent humoral response as an adjuvant treatment for RRP remains unknown [46]. One possible explanation could be that the underlying mechanism of recurrence is the result of incomplete removal of infected tissue actively producing virus and thereby causing local spread [47]. Therefore, the combination of complete surgical or chemical removal of existing lesions combined with the induction of sufficiently high antibody titers after vaccination and subsequent immunoglobulin secretion on the mucous membranes of the aerodigestive tract may help to prevent or delay recurrence [19, 46].

Because of that the authors think that the approach to combine an adjuvant HPV vaccination with surgical debridement and intralesional Cidofovir® injection is a promising treatment option for RRP.

## Conclusions

Our clinical results after adjuvant immunization of 13 patients show a positive effect on the course of the RRP. After vaccination, the recurrence rate appears to be significantly lowered compared to international literature.

For this reason, the authors believe that HPV vaccination in RRP has a beneficial therapeutic effect by preventing the formation of new papillomas and immunization may prevent a spread of the infection.

Further studies are needed to investigate the complete mechanism of this novel treatment approach.

## Abbreviations

HPV: Human papilloma virus
RRP: Recurrent respiratory papillomatosis
